# ‘When he’s up there he’s just happy and content’: Parents’ perceptions of therapeutic horseback riding

**DOI:** 10.4102/ajod.v6i0.307

**Published:** 2017-07-26

**Authors:** Lauren Boyd, Marieanna le Roux

**Affiliations:** 1 Department of Psychology, Stellenbosch University, South Africa

## Abstract

**Background:**

There is limited global and South African research on parents’ perceptions of therapeutic horseback riding (THR), as well as their perceptions of the effect of the activity on their children with disabilities.

**Objective:**

To explore and describe parents’ perceptions and experiences of THR as an activity for their children with disabilities.

**Method:**

Twelve parents whose children attend THR lessons at the South African Riding for the Disabled Association in Cape Town were asked to participate in a semi-structured interview. The qualitative data obtained from the interviews were first transcribed and then analysed using thematic analysis to establish parents’ perceptions of the THR activity.

**Results:**

The main themes that emerged included parental perceived effects of THR on children, parents’ personal experiences of the services, and parents’ perceived reasons for improvements in the children. The participating parents indicated that THR had had a positive psychological, social and physical effect both on the children participating in the riding, as well as on the parents themselves.

**Conclusion:**

According to parents, THR plays an important role in the lives of children with various disabilities and in the lives of their parents. The results of the study address the gap in the literature regarding parents’ perceptions of THR.

## Introduction and background

The domestication of animals occurred more than 12 000 years ago (All, Loving & Crane 1999), and since then humans and animals have had a longstanding beneficial relationship. From allowing psychiatric patients to care for animals as a replacement for restraints and drugs (Jalongo, Astorino & Bomboy 2004), to using as companion animals upon recommendation by Florence Nightingale for chronically ill patients (All et al. 1999), animals have shown enormous potential to help humans. These benefits have led to the use of animals in two main types of interventions: Animal-Assisted Activities (AAA) and Animal-Assisted Therapy (AAT) (Lentini & Knox 2009). The use of horses falls into both of these types of interventions, namely equine-assisted psychotherapy (EAP, a form of AAT), hippotherapy (HT, a form of AAT), and therapeutic horseback riding (THR, a form of AAA). EAP uses horses to obtain psychotherapeutic outcomes (Lentini & Knox 2009; Schultz, Remick-Barlow & Robbins 2007), which include improved self-esteem and self-confidence (Kersten & Thomas, as cited in Schultz et al. 2007). To facilitate EAP, a mental health professional and an equine professional are required to be present and involved in the therapy (Kruger & Serpell 2006).

With HT, a horse’s movement is used to rehabilitate a person’s physical or movement disorder, and a human services practitioner facilitates the process (Silkwood-Sherer et al. 2012). Although the main focus is on the physical effects (All et al. 1999; Kruger & Serpell 2006; Silkwood-Sherer et al. 2012; Tseng, Chen & Tam 2013), it does have psychological benefits for the client (Le Roux & Kemp 2009; Zadnikar & Kastrin 2011). THR takes place during a riding lesson taught by a riding instructor to a person with a disability or a chronic illness. The aim is to establish a therapeutic bond between the rider and the horse and to improve the quality of life of the rider (All et al. 1999; Bass, Duchowny & Llabre 2009; Silkwood-Sherer et al. 2012). Change takes place through interaction with the horse and by improving the functioning of the person with disabilities through the riding activity. THR was the focus of this study.

### Why therapeutic horseback riding?

Globally as well as within the South African context, literature on parents’ perceptions of THR and its effect on their disabled children, as well as their own personal experiences of the activity, is limited. The available literature, however, reveals beneficial experiences and effects of THR on children with disabilities (Davis et al. 2009; Miller & Alston 2004; Scialli 2002; Surujlal & Rufus 2011). There is limited South African research on parents’ perceptions of THR as an activity for their children with disabilities (Surujlal & Rufus 2011; Van Wyk 2014). THR has multiple physical, psychological and social effects, while at the same time disguising a therapeutic activity as an enjoyable experience. It brings together exercise and focus in a way that disguises its actual intention of assisting people with disabilities. THR has the potential to positively affect disabled children’s abilities to perform certain functions. The effects on certain physical, psychological and social functions are supported by Bream and Spangler (2001) and Scialli (2002) who stated that riding benefits people with disabilities mainly in these three areas. Britton (as cited in All et al. 1999) also stated that horseback riding for people with disabilities promotes physical, social and emotional healing.

Physical effects of THR include improvements in participants’ abilities to walk, run and jump (Cherng et al. 2009; Drnach, O’Brien & Kreger 2010; Low et al. 2005; Sterba et al. 2002). Gross motor function also improves following THR (Cuypers, De Ridder & Strandheim 2011; Scialli 2002; Winchester et al. 2002), and improvement is sustained following the end of participation in the activity. Postural control and balance (Bertoti 1988; Land, Errington & Paul 2002; Scialli 2002) as well as coordination (Brock 1990; Scialli 2002) is another set of physical functions that have been reported to improve following THR. Psychological functions, like a sense of accomplishment and achievement (All et al. 1999; Davis et al. 2009; Elliott, Funderburk & Holland 2008), resulting in increased self-confidence self-esteem, and self-worth, occurred following THR (All et al. 1999; Bass et al. 2009; Drnach et al. 2010; Lessick et al. 2004; Scialli 2002; Surujlal & Rufus 2011). Riding gives people with disabilities a chance to participate in and succeed at something that many people without a disability may hesitate to try (All et al. 1999; Lessick et al. 2004).

These psychological effects may in turn affect social functioning. A boost in confidence may lead to an increase in social participation (Debuse, Gibb & Chandler 2009). The participative environment and opportunity to interact with other children with similar disabilities also have a positive effect on social functioning (Lessick et al. 2004). Riding is a multisensory experience in which the participant is interacting with the horse as well as riding it (Bass et al. 2009), and communication with a horse may be less threatening as horses cannot speak back (Elliott et al. 2008). Hence, it was found that participants with autism spectrum disorders had improved communication skills following THR (Grandin, Fine & Bowers 2010). The majority of the literature reviewed focuses on the effect of THR on separate domains of functioning (i.e. physical, psychological or social), and only a few focus on the effect of THR on all domains of functioning (Davis et al. 2009; Elliott et al. 2008; Scialli 2002). This study did not focus on any specific effects of THR but rather aimed to gauge a general overview of the activity and its effects from the perspective of participating children’s parents.

## Methodology

### Research question

The following question guided the research and the data collection: What are parents’ perceptions and experiences of THR lessons for their children with disabilities?

### Research design

An exploratory qualitative research design was chosen for this study. It is appropriate as the aim was to explore parents’ perceptions of THR and not to restrict their answers and thoughts to a predefined set of experiences and views. A qualitative design provides a space for the investigation of an individual’s personal accounts of experiences and perceptions of themselves and the world around them (Merriam 2009).

### Participants

The research participants were parents of children with disabilities who participated in THR lessons at the South African Riding for the Disabled Association (SARDA) in Constantia, Cape Town. Convenience sampling was first chosen, as SARDA is an organisation that provides THR lessons to children with disabilities. Purposive sampling was then employed, which was based on the intended outcomes for the research (Lunenburg & Irby 2008). The participants had to meet the following criteria for inclusion in the study:

- A participant had to be a parent of a child who participated in THR lessons.
- The child had to be between the ages of 6 and 18 years. SARDA also teaches lessons to young adults over the age of 18. The wide age range was employed due to the small sample of parents who agreed to participate.
- The THR lessons had to take place at SARDA.
- Parents of children who did vaulting (movement on a stationary horse or barrel) were not included. The literature emphasised the importance of the movement of the horse and interaction with the live animal.

The first author spent the first week of the third school term of 2013 at SARDA, as well as another week in August. Every lesson was attended to obtain a large enough sample, and parents were invited to participate in the study if they so wished. Letters requesting participation from the parents, with the contact details of the author, were also given to the teachers to take home with them. This was done for those parents whose children come to the riding lessons by means of school transport. Twelve parents agreed to participate in an interview. Of the 12 participants, 11 were females and one was male. See Table 1 for demographic information on the parents, their children, the reasons for participation in THR and the length of time in the programme.

**TABLE 1:** Demographic information of participants.

Parent*	Child’s disability	Parents’ reasons for their children’s participation in THR	Time participating in THR
Kate	Prader–Willi syndrome	Physical, emotional, OT, social	6 months
Rachel	Cerebral palsy	Physical	6 months
Miriam	Cerebral palsy	Physical, recreational, OT	1 year
Hannah	Tuberous sclerosis	Physical, social, OT	5 years
Derek	Autism	Social, OT	1 year 7 months
Caitlin	Hearing and intellectual disability	Physical, OT	5 years
Sarah	Intellectual and physical disability	Medical, health	Many years
Rita	Left hemiplegia	Physical, emotional, recreation	13 years
Stella	Cerebral palsy	Physical, emotional, social, OT	13 years
Lindy	Cockayne syndrome	Physical, recreational	4 years
Megan	Down syndrome	Physical, emotional, social, OT	5 years
Sindy	Autism	Physical, OT, recreational	5 months

*Source*: Authors’ own work

THR, therapeutic horseback riding;

* , pseudonym; OT, occupational therapy.

### Data collection

Data were collected at SARDA in Constantia, Cape Town. Twelve parents volunteered to participate in a 30–45 min semi-structured interview at a time and place of their convenience. The interviews were completed over the period of December 2013–June 2014. The interviews lasted for about 15–70 min. A guide that was used in the interviews is included in Box 1. All interviews were audio-recorded with permission from the participants.

BOX 1 Questions used in the qualitative interviews.

- How did you and your child become involved with SARDA?
- Please elaborate on your child’s disability and their involvement at SARDA?
- Does your child enjoy the riding? How can you tell?
- Have you noticed any changes in your child since he or she started riding at SARDA? (Positive and negative)
- What has your experience been with horseback riding at SARDA?
- Would you recommend horseback riding as a mode of therapy to parents who have children with disabilities? Why and why not?

*Source*: Authors’ own work

### Data analysis

The audio-recordings were used to transcribe the interviews, and the transcriptions were analysed using thematic analysis and inductive reasoning. Thematic analysis searches for and analyses themes that occur more than once in the data set, and Braun and Clarke’s (2006) guidelines for thematic analysis were used by the author. The transcripts were thoroughly and repeatedly read and the author made notes to identify potentially important sections of the data. The data were then coded manually by identifying similar codes and patterns that could potentially make up a theme. Broad themes were narrowed down to more refined themes with different subthemes that were given names.

### Trustworthiness

To increase the trustworthiness of the study, a few strategies were followed. Peer examination by the first author’s supervisor as well as by external professionals with a background in psychology and research allowed for a fresh set of perspectives to be obtained (Shenton 2004). Following the peer examination, debriefing occurred in which the first author received feedback regarding vague sections in the research and potential biases to be aware of. Because of the first author being a horse rider, as well as having done personal research in the area previously, she needed to remain aware of her subjectivity in the study. To achieve reflexivity, self-awareness and reflection were consistently employed during the data collection and data analysis processes. A research journal was kept on days that the first author was physically at SARDA and approaching parents during lessons, and also on days that interviews were conducted with parents. This helped the first author to channel any personal experiences into the journal so as to not project them onto the research. Previous research findings on the topic were also reviewed to assess the congruency between the current results and previous results (Morrow 2005).

### Ethical consideration

Ethical approval was obtained from the Research Ethics Committee at Stellenbosch University (Ethics reference number DESC_Boyd2013). Signed informed consent from the parents involved in the study was also obtained before any data collection took place. Only parents were asked to participate in an interview. All participants and their children remained anonymous. Participation was voluntary, and the interviewees had the right to withdraw from the study at any time.

## Findings

Three main themes emerged from the data: (1) parents’ perceived effects of THR on their children with disabilities, (2) parents’ personal experiences of the service itself and (3) parents’ perceived reasons for improvements in their children.

### Parents’ perceived effects of therapeutic horseback riding on the children

Therapeutic horseback riding is a programme that brings about different perceived effects on the children participating – and not in one domain only. Most of the parents reported THR as an activity that has beneficial physical, psychological and social effects on their children, and no parents reported any negative effects of THR on their children.

### Physical effects

Physical effects are potentially the most noticeable effects on these children. Parents reported that THR had a big effect on their children’s posture and core stability. Most of the children with physical disabilities were initially unstable and limp on the horse and following involvement in the programme they could sit upright. As one participant described:

‘…With his physical, the physical side of it you can see. He’s more erect on the horse, he was quite floppy at first. So you can see that there’s …With the posture, his posture, there’s a significant improvement. He’s erect, he can control his body more.’ (Rachel, parent of Aaron, 6 years old, diagnosed with cerebral palsy)

Effects on the children’s muscles were also described. Lindy reported that her son had very tight muscles in his legs and was unable to walk ‘so it widens his legs up, which is great, which we wouldn’t normally be able to do at home’. Another parent had a child with low muscle tone and the THR had improved his muscle tone:

‘He had low muscle tone for a long time, and that’s improved enormously.’ (Stella, parent of Cara, 16 years old, diagnosed with cerebral palsy)

Strengthening of muscles leads to improvement in balance and the ability to ride alone. One mother reported that her child had begun THR lessons with two side walkers and now he no longer needs side walkers.

‘And you know, obviously, going from a leader and two side-walkers to just someone who’s leading the horse now, you know, all of that has been remarkable.’ (Stella, parent of Cara, 16 years old, diagnosed with cerebral palsy)

Improvement in the children’s muscles also leads to improvements in their walking abilities. Two parents reported that because of the THR there had been an improvement in their children’s abilities to walk:

‘Like my friends, and they see him, and they’re so shocked because he’s walking more, he can he used to take one, two steps. Now he … he can walk across this field.’ (Rachel, parent of Aaron, 6 years old, diagnosed with cerebral palsy)

‘We’ve gone from a child who’s not walking to a child who is … and gained some independence.’ (Miriam, parent of Nancy, 7 years old, diagnosed with cerebral palsy)

### Psychological effects

An important psychological aspect that the parents reported was the increase in confidence and independence of their children.

‘It gives them confidence and a sense of that they can control this big animal.’ (Derek, parent of Ben, 6 years old, diagnosed with autism)

‘I think the horseriding gives him a lot of independence. He can crawl so he does have a bit of independence, but … um … at horseriding I think he just. … I get the impression that he just feels like such a big boy.’ (Lindy, parent of Fred, 7 years old, diagnosed with Cockayne syndrome)

Pride and increased self-esteem were also reported by some of the parents:

‘Academically she can’t compete with any of them, her family members, but with the horseriding it gives her an edge. Cuz at least this is for her, it’s her own thing. So she’s special, cuz she’s the only one that does horseriding, and she’s doing well at it.’ (Rita, parent of Gemma, 16 years old, with left hemiplegia)

The children’s involvement in THR activities also brought about noticeable cognitive effects, such as skill-building, increased focus, and academic improvement.

‘And she is actually learning a skill, you know, she’s learning to master a skill. … So ya, I mean I would say it’s a big positive.’ (Kate, parent of Lily, 6 years old, diagnosed with Prader–Willi syndrome)

The involved nature of the activity, which requires the participants to concentrate and follow instructions, also noticeably led to improved focus, academic abilities, and comprehension, according to two parents.

‘She does become more focused.’ (Caitlin, parent of Jenny, 9 years old, with a hearing and intellectual disability)

‘It used to be quite difficult to explain stuff to her because her ability to her comprehension is so bad. And her comprehension has improved and… this is especially challenging to her because she’s having to comprehend from so many different people. … And I think that [her ability to follow instructions and do them] has definitely improved over the year.’ (Kate, parent of Lily, 6 years old, diagnosed with Prader–Willi syndrome)

A huge motivating factor in starting THR as well as continuing it was the joy and happiness parents had noticed their children receive from the activity. Parents mentioned the build-up to the riding lesson each week, and the happiness they saw in their children when at SARDA and around the horses.

‘She recognises where she’s going, and when she does she squeals with pleasure. And I generally have a squeal of pleasure as we drive up to SARDA, and so I know she’s looking forward to it.’ (Miriam, parent of Nancy, 7 years old, diagnosed with cerebral palsy)

The happiness was described as continuing once on the horse and riding:

‘When he’s up there he’s just happy and content.’ (Derek, parent of Ben, 6 years old, diagnosed with autism)

‘I mean she gets an absolute smile on her face as she gets on the horse, you know, most times, 9 out of 10 times.’ (Sarah, parent of Cindy, 17 years old, with an intellectual and physical disability)

It was also emphasised that the parents understood their children, and that they would definitely know if they were not enjoying the THR:

‘Now if she wasn’t … now I know enough that if she wasn’t interested in something, couldn’t care less, couldn’t exist. And it’s not like that; she’s definitely interested in it.’ (Hannah, parent of Angela, 15 years old, diagnosed with tuberous sclerosis)

Enrichment of their children’s lives in the form of a unique activity that benefited them in many different ways was an important aspect that many parents perceived as occurring as a result of THR. The parents interviewed regarded THR as an enjoyable activity for their children, spreading the joy and happiness into other aspects of their lives. They mentioned the fact that through THR their children were also spending time outdoors, exercising, and benefiting psychologically and physically:

‘I think for me the most important thing is that it’s a therapy she can benefit from, but she can relax while she’s doing it. She doesn’t realise it’s a therapy. Because you know these special needs kids, we sometimes forget about it but they have to work incredibly hard.’ (Caitlin, parent of Jenny, 9 years old, with a hearing and intellectual disability)

THR also places no limits on who can participate. Parents liked the fact that their children can participate in the activity, no matter what their disability:

‘I think … you know, the most noticeable thing is that there’s something that she does that she absolutely loves. … And also for her I think it’s so valuable because there’s not a lot of things that she can do that she’s gonna really be able to love, you know.’ (Kate, parent of Lily, 6 years old, diagnosed with Prader–Willi syndrome)

‘There’s not a lot of things that he can do so this is the one thing that he can do, so we’ll definitely carry on for as long as we can. … I think if he could horse ride everyday he would [*laughs*].’ (Lindy, parent of Fred, 7 years old, diagnosed with Cockayne syndrome)

The bond between the horses and the children was perceived as aiding the children in obtaining more benefit out of the activity:

‘If they can connect with the horse, love the horse, feel for the horse … it’s just a different dimension to the physiotherapy.’ (Rita, parent of Gemma, 16 years old, with left hemiplegia)

### Social effects

The involved nature of the activity encourages the development of social and behavioural skills, as mentioned by the parents. Parents expressed how their children’s speech has improved as well as their social confidence and ability to interact with other people.

‘Um … for people like with autism, it helps them with interacting with other individuals, even if that other individual is a horse.’ (Derek, parent of Ben, 6 years old, diagnosed with autism)

‘Building up a relationship with an animal is also something important for a child to learn I think, and also my daughter’s the only child so … I think that’s also nice for her to have that’ (Caitlin parent of Jenny, 9 years old, with a hearing and intellectual disability)

‘Um, then he started getting to a point of, um, social interaction, and it was all about the people that he was meeting, um and interacting with.’ (Stella, parent of Cara, 16 years old, diagnosed with cerebral palsy)

### Calming effects

The movement of the horse as a calming mechanism was mentioned by some parents. Some of their children had actually fallen asleep on the horse because the motion had been so relaxing. This effect was mentioned positively in relation to the children who have autism, attention-deficit hyperactivity disorder or sensory issues:

‘I recently once said to someone, if Jenny* could do everything she does on a horse she wouldn’t need Ritalin. It’s the only time of the week where she’s actually calm.’ (Caitlin, parent of Jenny, 9 years old, with a hearing and intellectual disability)

‘I think it’s also what they like, um, the kids with sensory issues like predictability, they don’t want sharp sudden sounds, they want something that’s predictable. And the movement of the horse is predictable, you know, it’s kind of calming.’ (Sarah, parent of Cindy, 17 years old, with an intellectual and physical disability)

### Parents’ experiences of the service itself

Parents commented not just on the perceived effects of THR on their children, but also on their own experiences of the service itself. They spoke about the environment, being given the opportunity of participating in the service, their satisfaction with the activity, as well as general feedback that they had.

### Uplifting environment

Parents acknowledged the uplifting and friendly environment in which the riding takes place, paying special mention to the actual environment, the staff, and the other parents who are in similar situations:

‘For me it was really a very safe haven as a parent with a disabled child.’ (Caitlin, parent of Jenny, 9 years old, with a hearing and intellectual disability)

‘So it’s nice when you are around other people in the same situation. You almost feel free.’ (Hannah, parent of Angela, 15 years old, diagnosed with tuberous sclerosis)

### Relief for the service

For many of the parents the THR service is one that helped them realise that they were not alone and options and opportunities existed for their children.

‘I was in the dark about everything cuz there was no-one to advise me, you know, except for the paediatrician. … So when I saw her on the horse that day I was so excited.’ (Sarah, parent of Cindy, 17 years old, with an intellectual and physical disability)

‘We’ve been rejected through a lot of things and they accept you. And they accept her [*daughter*].’ (Hannah, parent of Angela, 15 years old, diagnosed with tuberous sclerosis)

### Satisfaction for the parent

The happiness that the children gain from participating in the THR activity in turn brings about happiness in their parents. As one parent mentioned:

‘Before you know the time is up and we’re coming home, you know. And she’s happy. And then I know I’ve done good, you know.’ (Sarah, parent of Cindy, 17 years old, with an intellectual and physical disability)

### Feedback

Feedback from the parents during the course of the interviews related to participation in group classes, the number of times that their children were able to ride per week, and networking opportunities. Some parents felt that the group riding classes hindered their children’s abilities to improve their riding skills, and that the pace of getting the children ready to ride was quite slow:

‘So then as the instructor … you know, addressing the class, so Michael* and one girl got it in one [*clicks fingers*], and for the others, you know, the blinds come down. … Too much instruction.’ (Megan, parent of Diana, 17 years old, diagnosed with Down’s syndrome)

‘Cuz obviously with the group class everybody’s disabilities… they can’t really go ahead.’ (Rita, parent of Gemma, 16 years old, with left hemiplegia)

‘I used to get upset cuz you know, she takes forever to get the kids on, but you know, when she’s doing what … I mean she knows what she’s doing. You can’t hurry these things.’ (Sarah, parent of Cindy, 17 years old, with an intellectual and physical disability)

Some parents also mentioned that they would prefer if their children could ride more than once a week, as their children enjoyed it and it would be more effective. Constraints such as time, the number of volunteers, and the number of horses meant children were only able to ride once a week:

‘Look, riding once a week I think is not as effective as riding five times a week would be.’ (Miriam, parent of Nancy, 7 years old, diagnosed with cerebral palsy)

‘I mean if it was possible I would have taken her twice a week because also she’s good at it and because she likes it so much.’ (Caitlin, parent of Jenny, 9 years old, with a hearing and intellectual disability)

The parents emphasised their view that SARDA needed more networking. They spoke about how important the opportunity to ride at SARDA was for many children and families, and the fact that it was a free service. They mentioned that people wasted the opportunity and did not appreciate it:

‘I would say, if possible, SARDA need to network a lot more, and go and speak to all those therapists and … um … specialists, and tell them what they can do for their patients.’ (Stella, parent of Cara, 16 years old, diagnosed with cerebral palsy)

‘And I mean if it wasn’t for them a lot of kids wouldn’t have this opportunity. But there’s a lot of kids that have the opportunity and they’re not prepared to take it. … Nobody pays, you don’t pay a cent. Not one cent. And people waste it.’ (Rita, parent of Gemma, 16 years old, with left hemiplegia)

### Parents’ perceived reasons for improvement in their children

Parents differed about the reasons for their children’s improvement. Some parents felt there was a direct relationship between the improvements in their children and the THR activity. Other parents witnessed improvements in their children since participating in THR; however, they were not inclined to say it was a direct result of THR as they could not prove anything.

### Improvements due to a combination of circumstances and factors

Parents who were more inclined to say the improvements they had seen in their children were due to a combination of circumstances were referring to age-appropriate development and their children’s participation in a range of different therapies.

‘How do I measure the results? You can’t. … You can’t say that it’s necessarily because of speech therapy you know, or because of riding, but you know that there is improvement.’ (Sarah, parent of Cindy, 17 years old, with an intellectual and physical disability)

### Improvements due to therapeutic horseback riding

Despite believing that a combination of factors had led to improvements in their children, many parents emphasised their belief that THR had beneficial effects on their children.

‘So that’s how I know that the horseriding helps, because in between he’s getting his physio and that, but if he misses out on a big chunk of horseriding then, um, he definitely is stiffer and you battle to get his legs over the horse.’ (Lindy, parent of Fred, 7 years old, diagnosed with Cockayne syndrome)

## Discussion

The aim of the current study was to explore parents’ perceptions and experiences of their children’s involvement in THR. Twelve parents, each with a child participating in THR activities at SARDA, participated in this study. Three main themes emerged from the analysis of the interviews.

### Parents’ perceived effects of therapeutic horseback riding on children

Most parents reported on the noticeable physical effects the THR programme had had on their children. Some of the effects that were mentioned were improvement of posture, strengthening of muscles, changes in muscle tone, improvement in balance, and a change in walking abilities. The improvement in posture is supported by a study by Land et al. (2002) which showed via motion analysis equipment that after an 8–10-week riding programme, children involved showed improvement in their postural control. The children involved in Land et al.’s (2002) study had various disabilities. The parents in the present study reported strengthening of their children’s muscles, muscle tone and an improvement in balance, which confirmed the results of Bertoti (1988), who looked at the effects of THR on posture in children with cerebral palsy. Two of the parents interviewed in the present study stated that their children had improved walking abilities as a result of the THR programme. Cherng et al. (2009), Low et al. (2005), and Sterba et al. (2002), who studied THR and its effect on children with cerebral palsy, all reported improvements in participants’ abilities to walk, run and jump following THR.

Parents also reported on the changes in their children’s confidence, independence and pride following participation in the THR programme. The children’s confidence improved after being in contact with the large animal, which confirmed the results of Lessick et al. (2004) who found that controlling an animal of approximately 600 kg has a noticeable effect on improving confidence in the rider. Increased confidence, which leads to less fear of potentially painful movements for children with physical disabilities, is also reported in studies by Bertoti (1988), Davis et al. (2009), Drnach et al. (2010) and Sterba et al. (2002). In other South African studies, parents reported increased confidence in their children with disabilities, which led to more independence (Naidoo 2009; Surujlal & Rufus 2011). Children were able to communicate with the horse and they moved from a sense of powerlessness to experiencing success (Schultz et al. 2007). Children’s sense of achievement increases their motivation, which in turn benefits their motor abilities (Bartlett & Palisano 2002). The participants achieve success in an activity that is unique and challenging, even for many people who do not have disabilities (All et al. 1999). Cognitively, children became more focused and directional as a result of THR. In a study by Bass et al. (2009) autistic children displayed more focus and attention after participating in a 12-week THR intervention. When riding, the children are confronted with constant instructions and activities that they need to complete. Children in the current study also displayed improvements in speech and planning skills, confirming the results of Gabriels et al. (2012) who found significant improvements in children’s language and planning skills following a 10-week THR programme.

Parents reported an improvement in their children’s social functioning since attending the THR programme at SARDA. One parent reported that following interaction with the horse his autistic child was able to better interact with other individuals. This finding confirms that of Elliott et al. (2008) who asserted that communication with a horse might be easier for children with disabilities as the animal is non-judgmental. The bond that develops between the children and the horses allows them to develop qualities such as empathy, affection and confidence. Children in the THR programme improved their social confidence and enjoyed interaction with other children, as reported by their parents. This confirms results by Scialli (2002), Surujlal and Rufus (2011) and Weideman (2007) who all reported that the children in their studies (with a wide range of disabilities) were more engaging and interactive with other children following THR.

Parents mentioned that the movement of the horse relaxed and calmed their children, with some children so relaxed that they fell asleep. According to Stoner (as cited in Gabriels et al. 2012), as well as All et al. (1999), it is the warmth of the horse that brings about calm and relaxation for the children. The parents in the current study described their children as calmer, less anxious, and more positive on the days that they had their THR sessions. Parents in the current study also described their children as displaying joy leading up to their lessons at SARDA. They enjoyed being around the horses, and being part of the THR programme gave them the opportunity to do so. Other studies reported the same excitement and enjoyment of the children (Drnach et al. 2010; Scialli 2002). Enjoyment assists the children in pushing past barriers and discomfort caused by their disabilities (Lessick et al. 2004). According to the parents, the THR programme not only helped their children’s functional development but also their quality of life. Most of these children were involved in other therapies such as physiotherapy, speech therapy and occupational therapy. With THR, however, they could enjoy the activity (Elliott et al. 2008) which brings about an increase in interest and enjoyment in their lives (All et al. 1999). According to Grandin et al. (2010), THR is therapeutic due to the interaction with the horses and other people as opposed to interacting with just one person. Interactions with animals, as reported by Holen (2012), also lead to increased happiness, in turn raising the quality of a person’s life.

### Parents’ experiences of the service

Most parents spoke optimistically about the THR programme and being at SARDA. They had a good experience with the environment, the staff and other parents with whom they have built relationships. These results confirm those of Scialli (2002) in that parents whose children were participating in THR reported on the environment being peaceful. The parents in the current study praised the staff and the volunteers, as echoed by the parents in the study by Elliot et al. (2008). Parents have formed relationships with other parents and described SARDA as a place where they can relate to each other and make new friends in similar situations.

Parents were also grateful for the service from SARDA, which is a free service. They indicated that they would pay for such a service, as SARDA had been an invaluable part of their and their children’s lives. Parents in a study by Sterba et al. (2002) also confirmed that they would pay for a service like THR. Most parents were happy and excited about the THR programme. They as well as the parents in the studies done by Miller and Alston (2004) and Surujlal and Rufus (2011) expressed satisfaction and positivity about the THR programme.

In the feedback about the THR programme itself, specific mention was made of the group classes, the amount of time that the children are able to ride per week, and networking of SARDA. This type of feedback is important for the improvement of programmes (Elliot et al. 2008; Scialli 2002) as well as for potentially gaining funding (Scialli 2002). The gaining of funding is especially important for non-profit organisations such as SARDA and could potentially allow for more horses, more lessons and in turn the ability to assist more children with disabilities.

### Parents’ perceived reasons for improvements

The parents were divided in their opinions about the changes in their children. Some parents believed that it is a combination of factors and circumstances alongside THR that helped their children. The children were growing up, and developmental changes occurred. This comment is supported by Ntshangase (2008) who stated that from the age of 6 to 12 important cognitive, social and emotional developments occur. Furthermore, physical, cognitive and emotional developments occur from age 12 to adulthood (Shefer 2008). The children were also participating in additional therapeutic activities such as physiotherapy and speech therapy. Smith-Osborne and Selby (2010) presented the idea of THR as a complementary form of intervention, alongside conventional therapeutic activities, in assisting in the rehabilitation of disabilities, specifically in children and adolescents. Many parents still believed the THR had some effect on their children. One child was not involved in any other form of therapy while participating in THR, and some children showed adverse effects when they had a break from THR but still continued with other therapies. These findings confirmed those of Ward et al. (2013) who had found that the beneficial effects of THR in autistic children were not maintained after a break of six weeks from THR but returned once the riding began again.

## Limitations and future directions

The sample of the present study was homogenous, which could have limited the richness of the perceptions of the parents. Only parents whose children attended lessons at SARDA in the afternoon and parents who could receive the emailed request for participation from the stable manager were included. This, therefore, excluded potential economically disadvantaged participants. The sample size was also not large enough to reflect the South African population. Only 1 man and 11 women participated. These limitations need to be attended to in future research. A longitudinal study of children who have been participating in the riding programme for more than one year is advised. As mentioned in the study, participants’ children had been part of the riding programme for between 6 months and 13 years. A longitudinal study would potentially show that an extended period in the programme yields more beneficial and more specific outcomes than a shorter stint. A set of questions may also be too restrictive in eliciting parents’ experiences of THR for their children; it is advised that one or two open-ended questions guide future interviews in studies of this nature, allowing for more free flow of conversations, thoughts and experiences.

## Conclusion

The findings of the current study highlight the perceptions of parents whose children are involved in a THR programme at SARDA, a therapeutic riding association in South Africa. As seen from the results, parents perceived the THR programme as having played an important role in the lives of their children. The parents reported on the favourable effects in the physical, psychological and social domains. Furthermore, the parents believed their children gained enjoyment and that their quality of life improved. The results are supported by the existing literature. The study also contributed to narrow the gap in the literature on parents’ perceptions of their children’s involvement in THR – in South Africa as well as globally.

## References

[CIT0001] All A.C., Loving G.L. & Crane L.L, 1999, ‘Animals, horseback riding, and implications for rehabilitation therapy’, *Journal of Rehabilitation* 65, 49–57, viewed 19 February 2014, from http://equineassistedinterventions.org/pdf_dwn.php?tbl=papers&id=69&redir=/paper_display.php?page=15&order=Author2&sec=DESC.

[CIT0002] Bartlett D.R. & Palisano F, 2002, ‘Physical therapists perceptions of factors influencing the acquisition of motor abilities of children with cerebral palsy: Implications for clinical reasoning’, *Physical Therapy* 82, 237–248, viewed 20 April 2013, from http://www.ncbi.nlm.nih.gov/pubmed/11869152. 11869152

[CIT0003] Bass M.M., Duchowny C.A. & Llabre M.M, 2009, ‘The effect of therapeutic horseback riding on social functioning in children with autism’, *Journal of Autism & Developmental Disorders* 39, 1261–1267. https://doi.org/10.1007/s10803-009-0734-3 19350376 10.1007/s10803-009-0734-3

[CIT0004] Bertoti D.B, 1988, ‘Effect of therapeutic horseback riding on posture in children with cerebral palsy’, *Physical Therapy* 68, 1505–1512, viewed 25 February 2013, from http://ptjournal.apta.org/. 3174832

[CIT0005] Braun V. & Clarke V, 2006, ‘Using thematic analysis in psychology’, *Qualitative Research in Psychology* 3, 77–101. https://doi.org/10.1191/1478088706qp0630a

[CIT0006] Bream J.A. & Spangler W.Q, 2001, *Therapeutic horseback riding: An overview*, CATRA, The Capital Area Therapeutic Riding Association, viewed 15 March 2013, from http://www.catra.net/info/overview.html.

[CIT0007] Brock B.J, 1990, ‘Therapy on horseback: Psychomotor and psychological change in physically disabled adults’, *Resources in Education* 25, 5–27, viewed 10 May 2013, from http://eric.ed.gov/?id=ED313183.

[CIT0008] Cherng R.J., Liao H.F., Leung H.W.C. & Hwang A.W, 2009, ‘The effectiveness of therapeutic horseback riding in children with spastic cerebral palsy’, *Adapted Physical Quarterly* 21, 103–121, viewed 07 February 2013, from http://ir.lib.ncku.edu.tw/handle/987654321/80809.

[CIT0009] Cuypers K., De Ridder K. & Strandheim A, 2011, ‘The effect of therapeutic horseback riding on 5 children with attention deficit hyperactivity disorder: A pilot study’, *The Journal of Alternative and Complementary Medicine* 17, 901–908. https://doi.org/10.1089/acm.2010.0547 22010778 10.1089/acm.2010.0547

[CIT0010] Davis E., Davies B., Wolfe R., Raadsveld R., Heine B., Thomason P. et al., 2009, ‘A randomized controlled trial of the impact of therapeutic horse riding on the quality of life, health, and function of children with cerebral palsy’, *Developmental Medicine & Child Neurology* 51, 111–119. https://doi.org/10.1111/j.1469-8749.2008.03245.x 19191844 10.1111/j.1469-8749.2008.03245.x

[CIT0011] Debuse D., Gibb C. & Chandler C, 2009, ‘Effects of hippotherapy on people with cerebral palsy from the users’ perspective: A qualitative study’, *Physiotherapy Theory and Practice* 25, 174–192. https://doi.org/10.1080/09593980902776662 19384737 10.1080/09593980902776662

[CIT0012] Drnach M., O’Brien P. & Kreger A, 2010, ‘The effects of a 5-week therapeutic horseback riding program on gross motor function in a child with cerebral palsy: A case study’, *The Journal of Alternative and Complementary Medicine* 16, 1003–1006. https://doi.org/10.1089/acm.2010.0043 20809809 10.1089/acm.2010.0043

[CIT0013] Elliott S., Funderburk J.A. & Holland J.M, 2008, ‘The impact of the ‘Stirrup Some Fun’ therapeutic horseback riding program: A qualitative investigation’, *American Journal of Recreation Therapy* 7, 19–28, viewed 14 April 2013, from http://www.petpartners.org/document.doc?id=426.

[CIT0014] Gabriels R.L., Agnew J.A., Holt K.D., Shoffner A., Zhaoxing P., Ruzzano S. et al., 2012, ‘Pilot study measuring the effects of therapeutic horseback riding on school-age children and adolescents with autism spectrum disorders’, *Research in Autism Spectrum Disorders* 6, 578–588. https://doi.org/10.1016/j.rasd.2011.09.007

[CIT0015] Grandin T., Fine A.H. & Bowers C.M, 2010, ‘The use of therapy animals with individuals with autism spectrum disorders’, in Fine A.H. (ed.), *Handbook on animal-assisted therapy*, pp. 247–264, Elsevier, London, UK.

[CIT0016] Holen R, 2012, ‘The effects of animal interaction on happiness’, *The University of Minnesota Undergraduate Journal of Psychology* 7, 5–8, viewed 27 January 2014, from https://sites.google.com/a/umn.edu/sentience/.

[CIT0017] Jalongo M.R., Astorino T. & Bomboy N, 2004, ‘Canine visitors: The influence of therapy dogs on young children’s learning and well-being in classrooms and hospitals’, *Early Childhood Education Journal* 35, 9–16. https://doi.org/1082-3301/04/0800-0009/0

[CIT0018] Kruger K.A. & Serpell J.A, 2006, ‘Animal-assisted interventions in mental health: Definitions and theoretical foundations’, in Fine A.H. (ed.), *Handbook on animal-assisted therapy: Theoretical foundations and guidelines for practice*, pp. 21–34, Elsevier, London, UK.

[CIT0019] Land G., Errington Povalac E. & Paul S, 2002, ‘The effects of therapeutic riding on sitting posture in individuals with disabilities’, *Occupational Therapy in Health Care* 14, 1–12. https://doi.org/10.1080/J003v14n01_01 10.1080/J003v14n01_01 23931706

[CIT0020] Lentini J.A. & Knox M, 2009, ‘A qualitative and quantitative review of equine facilitated psychotherapy (EFP) with children and adolescents’, *The Open Complementary Medicine Journal* 1, 51–57, viewed 08 March 2013, from http://www.petpartners.org/document.doc?id=911

[CIT0021] Le Roux M.C. & Kemp R, 2009, ‘Effect of a companion dog on depression and anxiety levels of elderly residents in a long-term care facility’, *Psychogeriatrics* 9, 23–26. https://doi.org/10.1111/j.1479-8301.2009.00268.x

[CIT0022] Lessick M., Shinaver R., Post K.M., Rivera J.E. & Lemon B, 2004, ‘Therapeutic horseback riding: Exploring this alternative therapy for women with disabilities’, *Association of Women’s Health, Obstetric and Neonatal Nurses Lifelines* 8, 46–53, viewed 07 January 2014, from http://www.ncbi.nlm.nih.gov/pubmed/15031888. 10.1177/1091592304263956 15031888

[CIT0023] Low S., Collins G., Dhagat C., Hanes P., Adams J. & Fischbach R, 2005, ‘Therapeutic horseback riding: Its effects on gait and gross motor function in children with cerebral palsy’, *Scientific and Educational Journal of Therapeutic Riding* 11, 12–24, viewed 10 March 2013, from http://www.petpartners.org/document.doc?id=329.

[CIT0024] Lunenburg F.C. & Irby B.J, 2008, *Writing a successful thesis or dissertation: Tips and strategies for students in the social and behavioural sciences*, Corwin Press, Inc., Thousand Oaks, CA.

[CIT0025] Merriam S.B, 2009, *Qualitative research. A guide to design and implementation*, Jossy-Bass, San Francisco, CA.

[CIT0026] Miller J.H. & Alston A.J, 2004, ‘Therapeutic riding: An educational tool for children with disabilities as viewed by parents’, *Journal of Southern Agricultural Education Research* 54, 113–123, viewed 07 March 2013, from http://pubs.aged.tamu.edu/jsaer/pdf/Vol54/54-01-113.pdf.

[CIT0027] Morrow S.L, 2005, ‘Quality and trustworthiness in qualitative research in counselling psychology’, *Journal of Counselling Psychology* 52, 250–260. https://doi.org/10.1037/0022-0167.52.2.250

[CIT0028] Naidoo P, 2009, ‘An analysis of the experiences of children with cerebral palsy in therapeutic horse riding’, unpublished doctoral dissertation, University of Kwa-Zulu Natal, Durban, South Africa, viewed 10 May 2014, from http://oatd.org/oatd/record?record=handle%5C:10413%5C%2F7739.

[CIT0029] Ntshangase S, 2008, ‘Middle childhood’, in Swart L., de la Ray C., Duncan N. & Townsend L. (eds.), *Psychology: An introduction*, pp. 74–84, Oxford University Press, Cape Town, South Africa.

[CIT0030] Schultz P.N., Remick-Barlow G.A. & Robbins L, 2007, ‘Equine-assisted psychotherapy: A mental health promotion/intervention modality for children who have experienced intra-family violence’, *Health and Social Care in the Community* 15, 265–271. https://doi.org/10.1111/j.1365-2524.2006.00684.x 17444990 10.1111/j.1365-2524.2006.00684.x

[CIT0031] Scialli A, 2002, ‘Parent perceptions of the effectiveness of therapeutic horseback riding for children with disabilities’, unpublished doctoral dissertation, Lynn University, Boca Raton, FL, viewed 02 May 2013, from http://webcache.googleusercontent.com/search?q=cache:4yOHMyzoEaIJ:equineassistedinterventions.org/pdf_dwn.php%3Ftbl%3Dpapers%26id%3D294%26redir%3D/paper_display.php%3Fpage%3D15+&cd=2&hl=en&ct=clnk&gl=za.

[CIT0032] Shefer T, 2008 ‘Adolescence’, in Swart L., de la Ray C., Duncan N. & Townsend L. (eds.), *Psychology: An introduction*, pp. 74–84, Oxford University Press, Cape Town, South Africa.

[CIT0033] Shenton A.K, 2004, ‘Strategies for ensuring trustworthiness in qualitative research projects’, *Education for Information* 22, 63–75, viewed 08 September 2013, from http://www.crec.co.uk/docs/Trustworthypaper.pdf.

[CIT0034] Silkwood-Sherer D.J., Killian C.B., Long T.M. & Martin K.S, 2012, ‘Hippotherapy: An intervention to habilitate balance deficits in children with movement disorders: A clinical trial’, *Physical Therapy* 92, 707–717. https://doi.org/10.2522/ptj.20110081 22247403 10.2522/ptj.20110081

[CIT0035] Smith-Osborne A. & Selby A, 2010, ‘Implications of the literature on equine-assisted activities for use as complementary intervention in social work practice with children and adolescents’, *Child and Adolescent Social Work Journal* 27, 291–307. https://doi.org/10.1007/s10560-010-0201-1

[CIT0036] Sterba J.A., Rogers B.T., France A.P. & Vokes D.A, 2002, ‘Horseback riding in children with cerebral palsy: Effect on gross motor function’, *Developmental Medicine and Child Neurology* 44, 301–308. 12033715 10.1017/s0012162201002122

[CIT0037] Surujlal J. & Rufus S, 2011, ‘Perceptions of parents about equine therapy for children with intellectual disabilities’, *African Journal for Physical, Health Education, Recreation and Dance (AJPHERD)* 1, 372–385 viewed 10 June 2013, from http://reference.sabinet.co.za/sa_epublication_article/ajpherd_v17_supp2_a27.

[CIT0038] Tseng S., Chen H. & Tam K, 2013, ‘Systematic review and meta-analysis of the effect of equine-assisted activities and therapies on gross motor outcome in children with cerebral palsy’, *Disability and Rehabilitation* 35, 89–99. https://doi.org/10.3109/09638288.2012.687033 22630812 10.3109/09638288.2012.687033

[CIT0039] Van Wyk C, 2014, ‘Ouers se ervaring van deelnameaan ‘n perdondersteundeouerleidingsprogram’ [Parents’ experiences of participation in an equine-assisted parental guidance programme], *TydskrifvirGeesteswetenskappe* 54, 324–340.

[CIT0040] Ward S., Whalon K., Rusnak K., Wendell K. & Paschall N, 2013, ‘The association between therapeutic horseback riding and the social communication and sensory reactions of children with autism’, *Journal of Autism and Developmental Disorders* 43, 2190–2198. https://doi.org/10.1007/s10803-013-1773-3 23371511 10.1007/s10803-013-1773-3

[CIT0041] Weideman S, 2007, ‘Physically disabled adolescents’ experience of therapeutic riding: A phenomenological investigation’, unpublished master’s dissertation, North West University, Potchefstroom, South Africa, viewed 04 May 2013, from http://dspace.nwu.ac.za/handle/10394/9.

[CIT0042] Winchester P., Kendall K., Peters H., Sears N. & Winkley T, 2002, ‘The effect of therapeutic horseback riding on gross motor function and gait speed in children who are developmentally delayed’, *Physical and Occupation Therapy in Pediatrics* 22, 37–50. https://doi.org/10.1080/J006v22n03_04 12506820

[CIT0043] Zadnikar M. & Kastrin A, 2011, ‘Effects of hippotherapy and therapeutic horseback riding in postural control or balance in children with cerebral palsy: A meta-analysis’, *Developmental Medicine and Child Neurology* 53, 684–691. https://doi.org/10.1111/j.1469-8743.2011.03951.x 21729249 10.1111/j.1469-8749.2011.03951.x

